# Tissue-engineered 3-dimensional (3D) microenvironment enhances the direct reprogramming of fibroblasts into cardiomyocytes by microRNAs

**DOI:** 10.1038/srep38815

**Published:** 2016-12-12

**Authors:** Yanzhen Li, Sophie Dal-Pra, Maria Mirotsou, Tilanthi M. Jayawardena, Conrad P. Hodgkinson, Nenad Bursac, Victor J. Dzau

**Affiliations:** 1 Department of Biomedical Engineering, Duke University, NC 27708, USA; 2 Mandel Center for Hypertension Research and Duke Cardiovascular Research Center, Department of Medicine, Duke University Medical Center, Durham, NC 27710, USA.

## Abstract

We have recently shown that a combination of microRNAs, miR combo, can directly reprogram cardiac fibroblasts into functional cardiomyocytes *in vitro* and *in vivo*. Reprogramming of cardiac fibroblasts by miR combo *in vivo* is associated with improved cardiac function following myocardial infarction. However, the efficiency of direct reprogramming *in vitro* is relatively modest and new strategies beyond the traditional two-dimensional (2D) culture should be identified to improve reprogramming process. Here, we report that a tissue-engineered three-dimensional (3D) hydrogel environment enhanced miR combo reprogramming of neonatal cardiac and tail-tip fibroblasts. This was associated with significantly increased MMPs expression in 3D vs. 2D cultured cells, while pharmacological inhibition of MMPs blocked the effect of the 3D culture on enhanced miR combo mediated reprogramming. We conclude that 3D tissue-engineered environment can enhance the direct reprogramming of fibroblasts to cardiomyocytes via a MMP-dependent mechanism.

Cardiac injury in humans results in irreversible loss of cardiomyocytes followed by expansion of cardiac fibroblasts, collagen deposition, and formation of scar tissue^1^,^2^. Fibrotic remodeling of the injured myocardium negatively impacts contractility and electrical conduction, leading to further deterioration in heart function and eventual organ failure^2^. Converting cardiac fibroblasts within the scar tissue into cardiomyocytes is a promising strategy to improve heart function^3^,^4^. To that end, we, and others, have identified distinct sets of microRNAs and/or transcription factors that can directly reprogram cardiac fibroblasts into cardiomyocyte-like cells^5^,^6^,^7^,^8^,^9^,^10^. Importantly, microRNA and transcription factor mediated transdifferentiation of fibroblasts into cardiomyocytes promotes functional recovery of the infarcted heart^5^,^7^,^9^,^10^. However, the efficiency of direct reprogramming *in vitro* is relatively modest and new approaches beyond simple two-dimensional (2D) cell culture are needed to improve the speed and the extent of the reprogramming process. Furthermore, understanding the mechanism of enhanced direct reprogramming can inform the strategies to improve miR combo therapy *in vivo*.

Three-dimensional (3D) tissue-engineered cardiac patches have been shown to better mimic the native cardiac tissue environment compared to 2D cell cultures^11^,^12^. Hydrogels, cross-linked insoluble hydrophilic networks of water soluble polymers, are commonly used to form 3D engineered tissues as their biophysical properties can be precisely tailored^13^. For potential clinical use, functional cardiac tissue patches would be typically seeded with cardiomyocytes derived from human pluripotent stem cells^12^. However, these cell sources are associated with safety and ethical concerns, which may limit their human applications. Cardiomyocytes derived from directly reprogrammed fibroblasts would be clinically safer and more suitable for human cell therapy as they do not involve tumorigenic risks. It is currently unknown how 3D culture environments may affect the reprogramming of fibroblasts into cardiomyocytes.

We thus sought to explore the effects of a fibrin-based 3D culture environment^11^,^12^ on the direct miR combo reprogramming of cardiac fibroblasts into a cardiomyocyte fate. We show that culturing fibroblasts within a 3D fibrin-based hydrogel environment (“tissue bundle”) significantly improves the efficiency of direct cardiac reprogramming by miR combo as assessed by gene and protein expression of early and later cardiac differentiation markers. We further demonstrate that the improved cardiac reprogramming is mediated by the enhanced expression of MMPs in the 3D culture environment.

## Results

**Figure 1:**
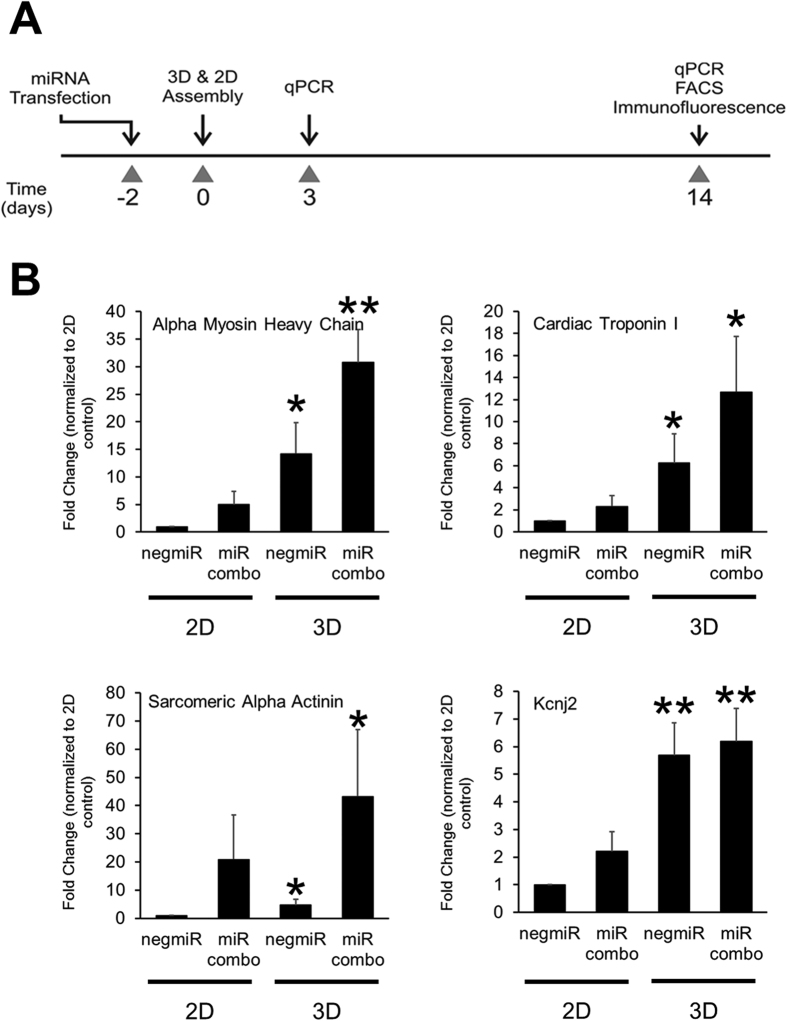
3D culture environment enhances miR combo mediated reprogramming at the genetic level. (**A**) Schematic of the experimental protocol. Neonatal cardiac fibroblasts were transfected with negative control miR (negmiR) or miR combo. Two days after transfection cells were re-plated either in regular culture dishes (2D) or encapsulated in a 3D hydrogel (3D). Cells were cultured for a further 14 days and cardiac gene expression analyzed by qPCR. (**B**) Comparisons of gene expression between 2D and 3D negmiR or miR combo groups (N = 5 independent transfections). *P < 0.05, **P < 0.005.

We have previously shown that a combination of microRNAs (miR-1, miR-133, miR-208, miR-499) that we termed miR combo, directly reprograms cardiac fibroblasts into cardiomyocytes both *in vitro* and *in vivo*^5^,^7^. We also showed that when seeded into 3D fibrin-based hydrogel tissues, cardiomyocytes derived from embryonic stem cells (ESCs), induced pluripotent stem cells (iPSCs) or neonatal heart tissues displayed enhanced structural and functional maturation^11^,^12^,^14^. Based on these studies, we hypothesized that culturing miR combo transfected fibroblasts within a similar tissue-engineered 3D environment would enhance reprogramming efficiency and provide mechanistic insights into the reprogramming process. To test this hypothesis, neonatal murine cardiac fibroblasts transfected with miR combo or the negative control microRNA (negmiR) were seeded on traditional tissue culture dishes (2D) or encapsulated in 3D hydrogels and 14 days later, the expression of cardiac genes was assessed by qPCR (Fig. 1A). We found that 3D culture environment significantly enhanced mRNA levels of the cardiac genes α-Myosin heavy chain (αMHC), Cardiac troponin-I, α-Sarcomeric actinin and Kcnj2 in both miR combo and negmiR control transfected neonatal cardiac fibroblasts (Fig. 1B).

**Figure 2:**
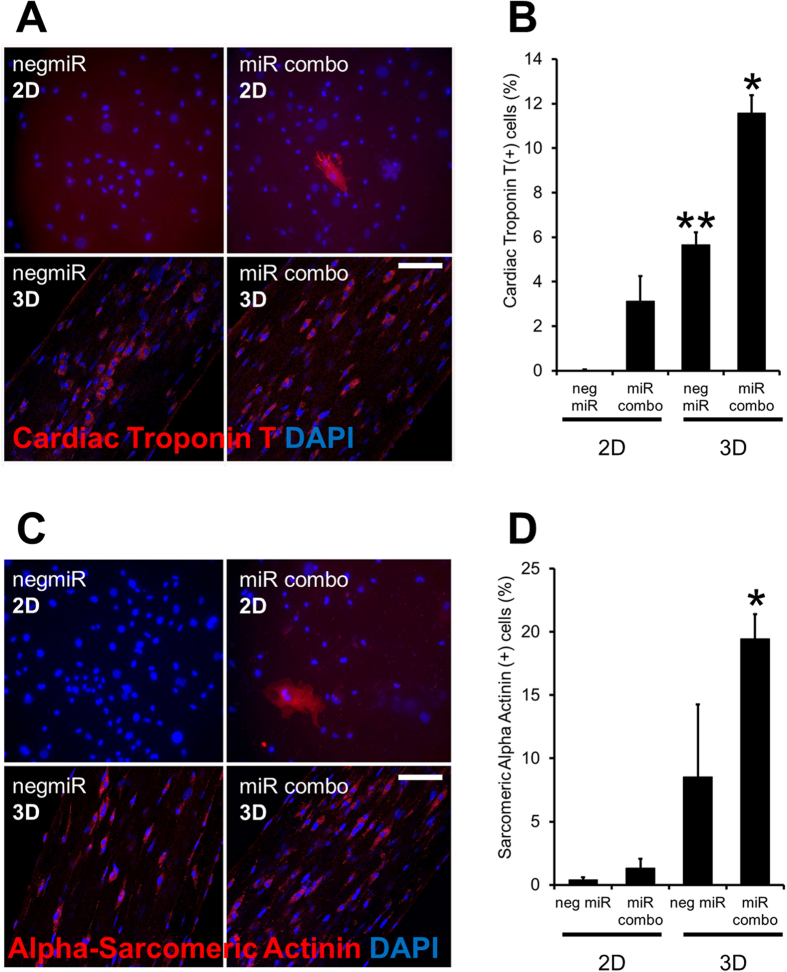
3D culture environment enhances miR combo mediated reprogramming at the protein level. Neonatal cardiac fibroblasts were transfected with negative control miR (negmiR) or miR combo. Two days after transfection cells were re-plated either in regular culture dishes (2D) or encapsulated in a 3D hydrogel (3D). Cells were cultured for a further 14 days. Cells were immunostained with antibodies for Cardiac troponin-T or α-Sarcomeric actinin. (**A**) Representative Cardiac troponin-T images with quantification provided in (**B**). (**C**) Representative α-Sarcomeric actinin images with quantification provided in (**D**). Comparisons made between respective 2D and 3D groups. **P < 0.001, *P < 0.01. N = 4 independent transfections. For each independent transfection two 3D bundles were stained. Scale bar 100 microns.

Immunostaining of wild-type cardiac fibroblasts cultured in 2D showed the expected increase in the number of Cardiac troponin-T(+) cells following miR combo treatment when compared to negmiR control group (Fig. 2A with quantification in Fig. 2B). Similar to the qPCR experiments, culturing cardiac fibroblasts in the 3D tissue bundles substantially increased the number of Cardiac troponin-T (+) cells in both the miR combo and negmiR control groups (Fig. 2A,B). This was also observed when cells were stained with α-Sarcomeric actinin (Fig. 2C with quantification in Fig. 2D). Interestingly, tissue bundles made of miR combo transfected fibroblasts displayed significantly lower stiffness compared to the negmiR control (Supplementary Figure 2), as is characteristic of healthy myocardial vs. fibrotic tissue.

**Figure 3:**
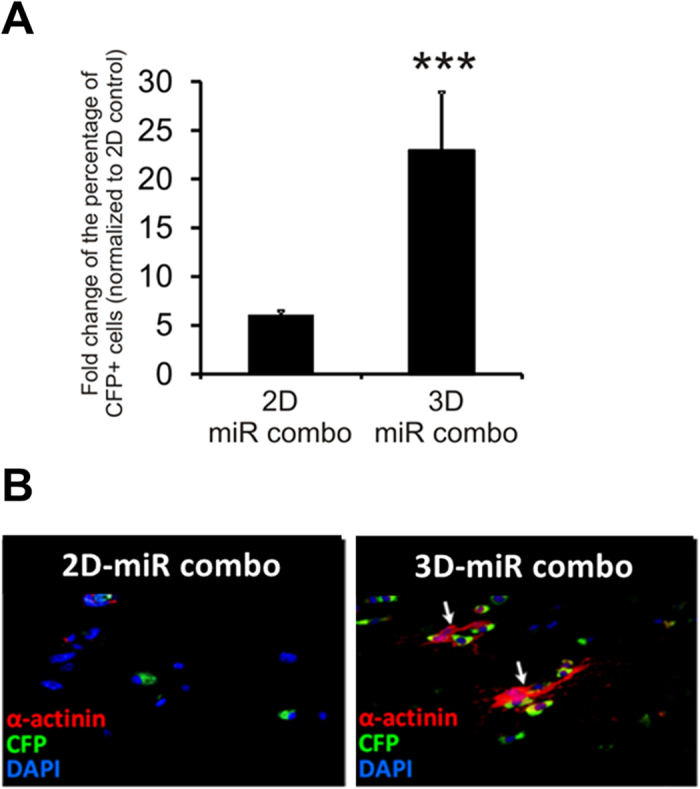
miR combo mediated induction of αMHC expression is enhanced by 3D culture environment. Representative FACS analysis (for CFP+ cells) and immunostaining images of neonatal cardiac fibroblasts from αMHC-CFP mice transfected with miR combo and cultured for 14 days in 2D or 3D environment (2D control were untransfected cells). N = 4 independent transfections. ***P < 0.001 between 2D and 3D.

To further assess reprogramming efficiency of miR combo transfected cells in 3D, we utilized the αMHC-CFP mouse model in which neonatal cardiac fibroblasts only express CFP following their transdifferentiation into cardiomyocytes^5^. As expected from our previous studies, miR combo induced reprogramming of fibroblasts into cardiomyocytes in 2D culture as evidenced by the ~5-fold increase in the number of CFP (+) cells (from FACS analysis) compared to untransfected control cells (Fig. 3A, 1.7% CFP(+) cells in control versus 7.8% CFP(+) cells for miR combo). In the 3D environment, efficiency of fibroblast reprogramming into cardiomyocytes in response to miR combo was substantially enhanced at 20-fold compared to untransfected control cells (Fig. 3A, 1.2% CFP(+) cells in control versus 23.1% CFP(+) cells for miR combo). The CFP (+) cells also expressed α-Sarcomeric actinin, more abundantly in 3D than 2D cultures (Fig. 3B). To further confirm that the newly formed Cardiac troponin-T (+) cells are derived from non-myocytes, we utilized the mouse genetic lineage-tracing model in which fibroblasts and their progeny were permanently labeled with the fluorescent protein tdTomato under the control of Fsp1 promoter^5^. This model confirmed the fibroblast origin of Cardiac troponin-T (+) cells in both 2D and 3D cultures (Supplementary Figure 3). Furthermore, whilst we confirmed the presence of tdTomato (+)/Cardiac troponin-T (+) cells in the miR combo treated group in 2D, we found that the number of double positive cells was dramatically increased in 3D. Interestingly, akin to our findings at the mRNA level, culturing neonatal cardiac fibroblasts in the 3D environment was sufficient to promote cardiac protein expression irrespective of the miR combo treatment (Supplementary Figure 3).

**Figure 4:**
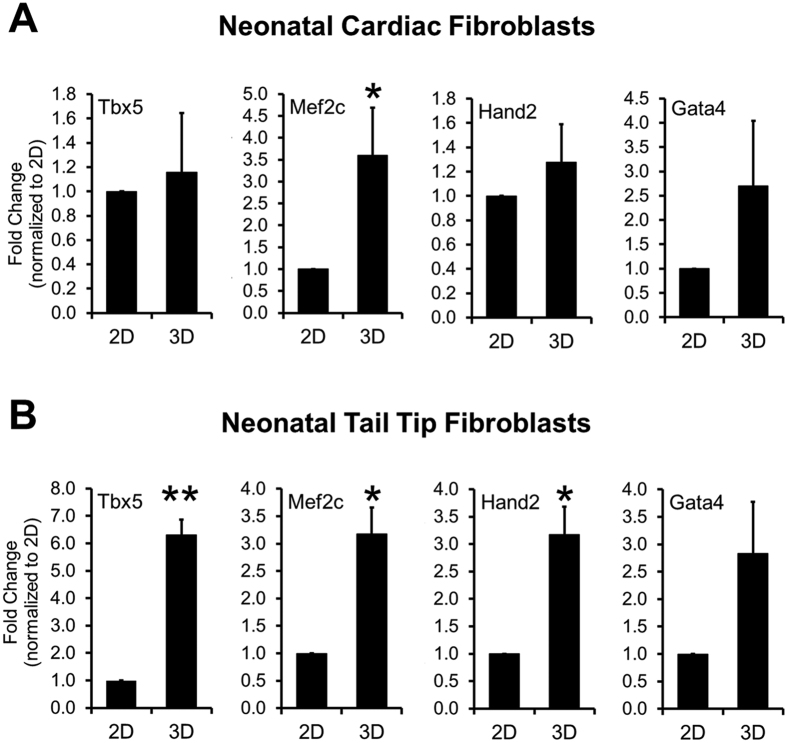
3D culture environment enhances expression of early cardiac transcription factors in fibroblasts. Expression of early cardiac transcription factors in neonatal cardiac (**A**) or tail-tip (**B**) fibroblasts cultured for 3 days in 2D or 3D environment (N = 3 independent transfections). *P < 0.05, **P < 0.005 between 2D and 3D.

Activation or increased expression of the early cardiac transcription factors Mef2C, Gata4, Tbx5 and Hand2 is known to promote reprogramming of fibroblasts into cardiomyocytes as evidenced by their induced expression of Cardiac troponin-T, α-Myosin heavy chain and α-Sarcomeric actinin^5^,^6^,^15^. Consequently, we wanted to determine if the enhancement of Cardiac troponin-T, α-Myosin heavy chain and α-Sarcomeric actinin expression in the 3D vs. 2D culture environment was dependent upon the activation of cardiac transcription factors at early stages. We found that 3D environment significantly up-regulated Mef2C expression at culture day 2, while the expression levels of Gata4, Hand2 and Tbx5 were similar in 3D vs. 2D cultured cardiac fibroblasts (Fig. 4A). Similar results were obtained with neonatal tail-tip fibroblasts with significant effects also found for expression of Tbx5 and Hand2 (Fig. 4B). Overall, these results suggested that the enhanced expression of cardiac proteins such as Cardiac troponin-T and α-Sarcomeric actinin in the 3D environment likely involved additional mechanisms beyond the robust induction of cardiac transcription factors.

**Figure 5:**
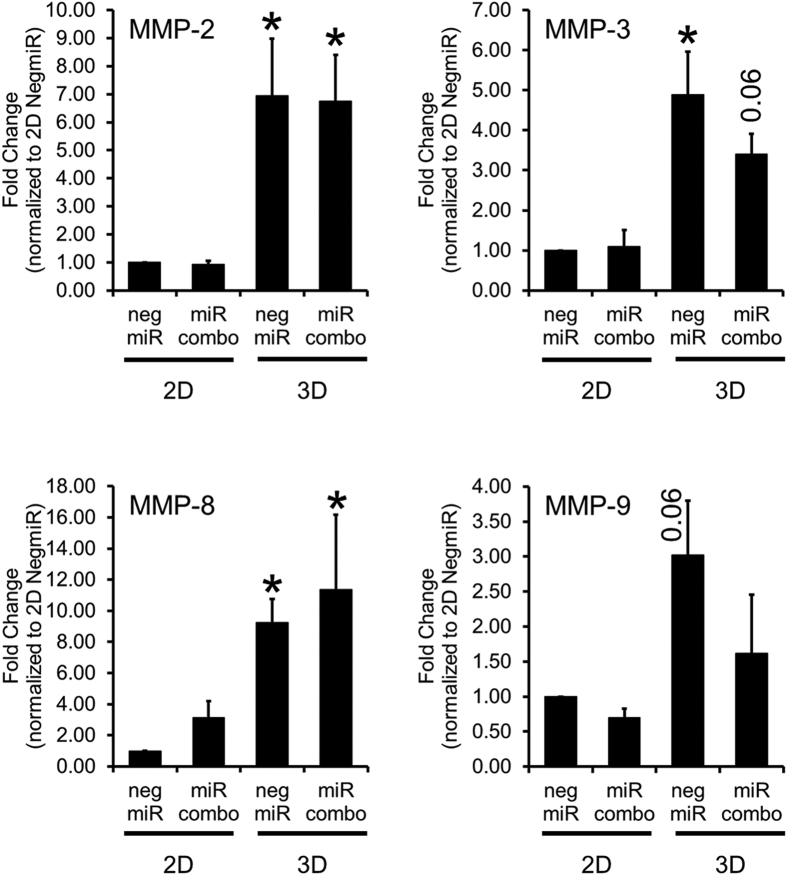
3D environment enhances MMP expression. Gene expression of different MMPs in neonatal mouse cardiac fibroblasts transfected with negative control miR (negmiR) or miR combo and cultured for 14 days in 2D or 3D environment (N = 3–4 independent transfections). P-values are indicated as shown or represented by *for a P-value of less than 0.05. Comparisons made between 2D and 3D.

**Figure 6:**
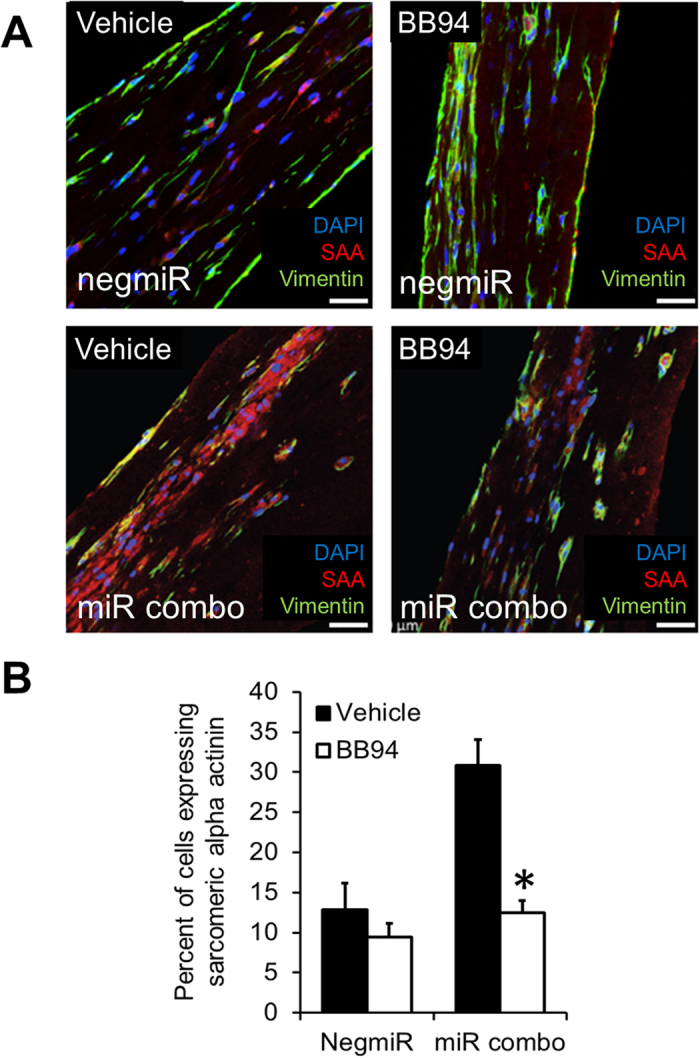
Effects of 3D environment upon miR combo treatment are MMP-mediated. Neonatal cardiac fibroblasts were transfected with negative control miR (negmiR) or miR combo. Two days after transfection cells were re-plated either in regular culture dishes (2D) or encapsulated in a 3D hydrogel (3D). Cells were cultured for a further 14 days in the presence of vehicle or 5 uM BB94 (a broad spectrum MMP inhibitor). Cells were immunostained with antibodies for α-Sarcomeric actinin. (**A**) Representative images, scale bar 100 microns, (**B**) Quantification, N = 3 independent transfections. For each independent transfection two 3D bundles were stained.*P < 0.05 between BB94 treatment and control in miR combo group.

We then explored potential mechanisms by which the 3D tissue bundle environment enhanced the reprogramming ability of miR combo. We performed a qPCR array assessing the expression of 84 genes important for cell-cell and cell-matrix interactions in miR combo transfected cardiac fibroblasts cultured in 2D or 3D environment. Several matrix metalloproteinases (MMPs) exhibited increased expression in 3D compared 2D cultured miR combo transfected fibroblasts (Supplementary Figure 4). Based on these results and importance of MMPs for cardiac remodeling in post-infarction disease^16^,^17^,^18^,^19^, we decided to focus our studies on the potential roles of MMPs in cardiac reprogramming. We found that culturing neonatal cardiac fibroblasts in 3D, but not 2D, strongly induced MMP expression (Fig. 5). MMP-2 and MMP-3 (Fig. 5) were the more strongly expressed MMPs in 3D and their expression was similar between the control negmiR and miR combo groups (Fig. 5). MMP-8 and MMP-9 mRNA levels showed a similar trend with significance reached for MMP-8 only (Fig. 5). A broad spectrum MMP pharmacological inhibitor BB94 was then used to determine if the increase in MMP activity could be causative of the effect of the 3D environment upon cardiac protein expression. Specifically, BB94 which is also known as Batimastat, inhibits MMP-1 (IC50: 3 nM), MMP-2 (IC50: 4 nM), MMP-3 (IC50: 20 nM), MMP-7 (IC50: 6 nM) and MMP-9 (IC50: 4 nM)^20^. In vehicle treated cells, miR combo significantly increased the reprogramming of fibroblasts into cardiomyocytes as evidenced by the 2-fold increase in the number of α-Sarcomeric actinin(+) cells when compared to the negmiR control group (Fig. 6, p = 0.03). This effect was abolished by BB94 (Fig. 6), suggesting that MMP activity is necessary for the enhanced cardiac reprogramming of fibroblasts observed in the 3D environment. Similar results were obtained at the mRNA level (Supplementary Figure 6).

## Discussion

In this study we show the first evidence that a tissue-engineered 3D hydrogel environment enhances miR combo mediated reprogramming of fibroblasts to cardiomyocytes and that MMP activity contributes to this process. Mechanistic details of how fibroblasts are directly converted into cardiomyocytes, either by overexpression of transcription factors or miRNAs, are lacking. On the other hand, it is well established that the direct reprogramming of cardiac fibroblasts in infarcted hearts *in vivo* gives rise to a much higher number of mature cardiomyocytes over a significantly shorter time compared to *in vitro* culture^9^,^21^,^22^. We thus reasoned that use of 3D cell culture to approximate *in vivo* environment^12^,^23^,^24^,^25^ could both improve cardiac reprogramming efficiency *in vitro* and provide additional insights into the reprogramming process. With the continued advances in tissue-engineering field, 3D cell culture environments of increasing complexity have been developed to overcome limitations of conventional 2D cultures that do not faithfully reproduce the structure and composition of extracellular matrix, concentrations of soluble factors, mechanical signals, or cell-cell communication present in native tissues^26^,^27^. Since these signaling cues play critical roles in regulating cell differentiation and function^28^,^29^,^30^,^31^,^32^, it is not surprising that 3D culture systems have promoted maturation of primary or stem cell-derived cardiomyocytes^12^,^33^,^34^; yet, until now, they have not been applied to study a distinct process of the direct microRNA reprogramming of fibroblasts into cardiomyocytes. The current study is the first to demonstrate that a 3D tissue-engineered environment can promote the direct miRNA reprogramming of fibroblasts into cardiomyocytes and that MMPs play important roles in the reprogramming process.

In our studies, MMP expression was significantly upregulated in 3D compared to 2D cell culture environment and this might have preconditioned fibroblasts to be more amenable to direct cardiac reprogramming. More importantly, inhibition of MMP activity through the pharmacological inhibitor BB94 abrogated the effect of 3D culture upon miR combo reprogramming. Based on these results, it is tempting to speculate that the known acute upregulation of MMPs in infarcted hearts^16^,^17^,^18^,^19^ might be one of the contributing factors to more successful direct reprogramming outcomes compared to *in vitro* studies. Consistent with our findings, other studies have suggested a role for MMPs in analogous processes such as cardiac differentiation of pluripotent stem cells. Specifically, Chung *et al*. showed that increased activity of MMP was required for spontaneous cardiomyocyte differentiation from mouse embryoid bodies embedded in 3D hydrogels, while treatment with the broad-spectrum MMP inhibitor PD166793 delayed the differentiation process^35^. Moreover, MMP-3 upregulation was found to be associated with Noggin-mediated cardiac lineage commitment of mouse ESCs, with MMP-3 knockdown leading to decreased expression of cardiac markers and cardiomyocyte yield^36^. Importantly, identifying and targeting the MMPs involved in the reprogramming process could enhance the efficiency and improve therapeutic outcomes of miRNA reprogramming *in vivo* and potentially lead to discovery of small reprogramming molecules that would obviate need for miRNA transfection.

The field of direct cardiac reprogramming is still in its infancy and it is unclear if gene or cell therapies will provide the optimal therapeutic approach. Currently, viral delivery of reprogramming factors into the heart has attracted the most attention; however, the lack of cell specificity and the low transduction efficacy of viruses may eventually preclude their clinical use. *In vitro* reprogramming of human fibroblasts, from any tissue origin, if highly efficient, may provide an alternative source of functional cardiac muscle cells in an expedited and tumor-free fashion, a clear advantage over the use of iPSC technology. Our 3D tissue-engineered model has incorporated only some of the cues present in native myocardium. However, more complex cell culture conditions could potentially offer an opportunity to further enhance the effect of miR combo in a 3D environment *in vitro*. In particular, hydrogels could be designed to incorporate small peptide enhancers of direct reprogramming as well as mechanical signals^37^. In fact, stem cell differentiation is regulated by both the cell traction forces that are generated via cell-mediated degradation of a hydrogel and the viscoelasticity of the hydrogel. For example, high cell traction favors osteogenic differentiation of human mesenchymal stem cells while low cell traction favors adipogenesis^38^. Similarly, rapidly relaxing hydrogels promote osteogenesis while suppressing adipogenesis^39^. It is thus plausible that incorporating these and other biomimetic cues in 3D cell cultures could further improve the cardiomyocyte yield.

Finally, the relative ease with which both cardiac and tail-tip fibroblasts were driven to express cardiac genes *in vitro* in this and a number of previous studies, suggests that these cells possess an inherent plasticity. Interestingly, in our study the 3D fibrin-based hydrogel environment alone was sufficient to enhance expression of cardiac program genes in fibroblasts. Previously, 3D sphere cultures of mouse fibroblasts have been shown to facilitate formation of neuronal progenitor cells without any genetic manipulations^40^. Furthermore, fibroblasts encapsulated in 3D hydrogels spontaneously expressed osteopontin and exhibited pre-osteogenic phenotype, and better responded to osteogenic differentiation compared to 2D cultures^41^. Together, these findings support the idea that the 3D *in vitro* environment on its own may affect the plasticity of somatic cells.

In summary, we have shown that 3D cell culture environment can enhance cardiac reprogramming of fibroblasts by miRNAs via MMP-mediated mechanisms. These findings are important first step towards the future use of tissue-engineered strategies to aid fibroblast targeted therapies for regeneration of infarcted heart.

## Methods

### MicroRNA transfection

Neonatal cardiac and tail-tip fibroblasts were isolated as described previously^5^,^6^,^7^. Fibroblasts were seeded into T150 flasks at 4,500 cells per cm^2^. After 24 hours, the cells were transfected with the non-targeting microRNA negmiR (Pre-miR™ miRNA Precursor Negative Control #1, Ambion) or with our previously reported combination of cardiac reprogramming microRNAs named miR combo^5^ (pre-miR^TM^ miRNA precursors for miR-1, miR-133, miR-208, miR-499, Ambion) using Dharmafect I (Thermo Scientific) transfection reagent. The transfection method has been previously described by Jayawardena *et al*.^7^. Two days after transfection cells were removed by 0.05% (w/v) trypsin, counted and seeded onto 24-wells tissue culture plates (20,000 cells per well) or into 3D hydrogels (150,000 cells per bundle).

### Fibroblast culture in 3D engineered tissue bundles

Fibroblasts were encapsulated into cylindrical 3D hydrogel constructs (tissue bundles) by modifying our previously published methods for engineering muscle tissues^11^,^12^,^21^,^42^,^43^ (Supplementary Figure 1). Briefly, 150,000 fibroblasts were mixed in 20 μl of culture media (DMEM, 10% (v/v) horse serum, 1% (v/v) chick embryo extract, 100 U/ml penicillin G, 1 mg/mL Aminocaproic Acid and 50 μg/ml Ascorbic Acid) and 20.4 μl of hydrogel solution (8 μl of 1 mg/ml Fibrinogen (Akron) +4 μl of Matrigel +8 μl of 2x media +0.4 μl of 50 unit/ml thrombin in 0.1% BSA in PBS) on ice. The cell/hydrogel mixture was pipetted into polydimethylsiloxane (PDMS) molds cast from Teflon masters that were placed in 12-well plates. The molds were pre-treated with 0.2% (w/v) pluronic (Invitrogen) and fitted with laser-cut Cerex^®^ frames (9.2 × 9.5 mm outer dimensions, 6.8 × 8.3 mm inner dimensions). The cell/hydrogel mixture was polymerized within PDMS molds for 45 min at 37 °C followed by addition of 2 ml of culture media per well. Frames with polymerized tissue bundles were removed from the molds the next day and cultured dynamically in suspension for additional 2 or 13 days. Media was changed every other day. For the BB94 experiments, bundles were treated with either 5 μM BB94 or DMSO (vehicle) at the day of bundle setup. Media was changed every day with fresh BB94 or DMSO containing culture media.

### Animal models

αMHC-CFP and Fsp1-Cre/tdTomato mice have been previously described^5^. All experiments using animal models were performed in accordance with institutional guidelines (DLAR, Duke University Division of Laboratory Animals, and IACUC, American Association for Laboratory Animal Science). All experimental protocols were approved by IACUC prior to the experiments being carried out.

### qPCR

Total RNA was extracted using RNeasy Fibrous Tissue Mini Kit and RNeasy Plus Mini Kit according to the manufacturer’s instructions (Qiagen). Total RNA was converted to cDNA using a high capacity cDNA reverse transcription kit (Applied Biosystems). cDNA was used in a standard qPCR reaction involving FAM conjugated gene specific primers and TaqMan Gene Expression Master Mix (Applied Biosystems). The following primers were used for qPCR: Gapdh (Mm99999915_m1), Tnni3 (Mm00437164_m1), Actn2 (Mm00473657_m1), Myh6 (Mm00440359_m1), Kcnj2 (Mm00434616_m1), Tbx5 (Mm00803518_m1), Mef2C (Mm01340842_m1), Gata4 (Mm00484689_m1), Hand2 (Mm00439247_m1), MMP-2 (Mm00439498_m1), MMP-3 (Mm00440295_m1), MMP-8 (Mm00439509_m1) and MMP-9 (Mm00442991_m1).

### qPCR array

RT^2^ Profiler PCR Array was performed according to the manufacturer’s instructions (Qiagen).

### Immunofluorescence and image analysis

2D cultured cells and tissue bundles were fixed with 2% (v/v) paraformaldehyde (EMS) on a rocking platform at room temperature for 10 min or at 4 °C overnight respectively. Fixed samples were blocked in antibody buffer (1% (w/v) BSA, 0.3% (v/v) Triton X-100, in PBS) for 1 hour at room temperature and then incubated with primary antibodies overnight at 4 °C in antibody buffer. Primary antibodies for α-Sarcomeric actinin (Sigma A7811, 1:200 dilution) and Cardiac troponin-T (Thermo Scientific MS-295-P0, 1:200 dilution) were used at indicated concentrations. Alexa-Fluor conjugated secondary antibodies (Invitrogen) were used at a 1:1000 dilution in antibody buffer for 2 hours at room temperature. Nuclei were stained with DAPI at 1 μg/ml for 30 minutes at room temperature in PBS. 2D cultures were imaged using a Zeiss Axiovert 200 fluorescent microscope. For each 2D condition (negmiR or miR combo), we stained 2 wells per antibody and we acquired a minimum of 5 images per well. For 2D experiments, we averaged the 5+ images taken from each well. Bundles were washed in PBS, and then mounted on slides and imaged 10–20 μm underneath the bundle surface using a Leica inverted SP5 confocal microscope. We acquired a minimum of 3 images per bundle. We stained two 3D bundles for each independent transfection and values were not averaged for each bundle. Images from 2D and 3D cultures were analyzed by ImageJ software.

### Flow cytometry

Neonatal cardiac fibroblasts derived from αMHC-CFP mice were isolated as described above. Fibroblasts were seeded into T150 flasks at 4,500 cells per cm^2^ and 24 hours later transfected with miR combo as described above. Two days after transfection cells were removed by 0.05% (w/v) trypsin, counted and re-plated either in regular culture dishes (2D: 20,000 cells per well) or encapsulated in a 3D hydrogel (3D: 150,000 cells per bundle). Cells were cultured for a further 14 days after which they dissociated. For 2D cultures this involved incubating the cell layer with 0.05%w/v trypsin for 5 minutes at 37 °C. For 3D cultures, the bundles were incubated with 0.25%w/v trypsin for a total of 45 minutes (three 15 minute incubations with fresh trypsin) at 37 °C. FACS was performed with a FACStar Plus cytometer (BD Biosciences) instrument configured with a CFP-specific laser. Untransfected cells cultured in regular culture dishes (2D) served as the control.

### Measurement of passive tension

Viscoelastic properties of the 3D tissue bundles were measured as previously described using a custom-made apparatus consisting of an ultra-sensitive optical force transducer coupled to a computer-controlled linear actuator^11^,^12^,^42^,^44^. Briefly, the tissue bundle was transferred into a measurement chamber filled with 37 °C Tyrode’s solution. The bundle was mounted at its culture length and baseline passive tension (F_base_) was measured. The two lateral sides of the Cerex frame were then removed and linear actuator was driven by computer to stretch the bundle in 4% strain increments (0–24% strain). Passive tension was measured at each strain (F_strain_) and difference (F_strain_ − F_base_) determined and plotted using a custom MATLAB program.

### Statistics

Results are presented as mean ± SEM. Statistical significances were evaluated by paired t-tests (one tail). P < 0.05 was considered statistically significant.

## Additional Information

**How to cite this article**: Li, Y. *et al*. Tissue-engineered 3-dimensional (3D) microenvironment enhances the direct reprogramming of fibroblasts into cardiomyocytes by microRNAs. *Sci. Rep.* **6**, 38815; doi: 10.1038/srep38815 (2016).

**Publisher's note:** Springer Nature remains neutral with regard to jurisdictional claims in published maps and institutional affiliations.

## Supplementary Material

Supplementary Information
