# Supplementary material for: Tissue-engineered 3-dimensional (3D) microenvironment enhances the direct reprogramming of fibroblasts into cardiomyocytes by microRNAs
Source: Sci Rep. 2016 Dec 12;6:38815. doi: 10.1038/srep38815 (PMC5150639; doi:10.1038/srep38815)

SUPPLEMENTARY

Tissue-engineered 3-dimensional (3D) microenvironment enhances the direct reprogramming of fibroblasts into cardiomyocytes by microRNAs.

Yanzhen Li, Sophie Dal-Pra, Maria Mirotsou, Tilanthi M. Jayawardena, Conrad P. Hodgkinson, Nenad Bursac, Victor J. Dzau

Supplementary Figures

Supplementary Figure 1. Design of the 3D tissue bundles. (A) Fibroblasts were mixed in a fibrin/matrigel matrix and then cast into wells of PDMS molds and anchored to nylon frames. After one day of compaction, frames with bundles were removed for free-floating culture. (B) Representative live images of 2-week old bundles with fibroblasts transfected with either Neg miR or miR combo. (C) Quantification of bundle diameters (N=7). **** $P < 0.0001$.

Supplementary Figure 2. miR combo reduces passive force in 3D cultures. Passive force-length relationships in 3D tissue bundles made of fibroblasts transfected with negative control miR (negmiR) or miR combo and cultured for 14 days (N=3). * $P<0.05$ between negmiR and miR combo. Passive force-length of a neonatal rat ventricular myocytes (NRVMs) enriched population is provided by way of comparison.

Supplementary Figure 3. miR combo reprogramming in Fsp1+ fibroblasts. (A) Representative immunostaining images of neonatal mouse cardiac fibroblasts derived from Fsp1Cre:tdTomato mice transfected with miR combo and cultured for 14 days in 2D or 3D environment. (B) Quantitative comparison of cTnT/TdTomato and SAA/TdTomato expressing

cells in 2D and 3D culture. * $P<0.05$, ** $P<0.01$. Comparisons made between 2D and 3D groups.

Supplementary Figure 4. **Gene expression in 3D vs. 2D culture environment.** Expressions of 84 genes important for cell-cell and cell-matrix interactions were assessed using a qPCR array (RT² Profiler™ PCR Array Mouse Extracellular Matrix & Adhesion Molecules, QIAGEN) in 14-day 2D and 3D cultures of miR combo transfected cardiac fibroblasts. Table and graph show the genes with significantly ($p<0.05$) upregulated expression in 3D compared to 2D environment.

Supplementary Figure 5. MMP inhibition blocks the effect of the 3D environment upon cardiac reprogramming. Neonatal cardiac fibroblasts were transfected with negative control miR (negmiR) or miR combo. Two days after transfection cells were re-plated either in regular culture dishes (2D) or encapsulated in a 3D hydrogel (3D). Cells were cultured for a further 14 days in the presence of vehicle or 5 uM BB94 (a broad spectrum MMP inhibitor). Cardiac gene expression was analyzed by qPCR. N=3, comparisons were not significant.

Supplementary Figure 1

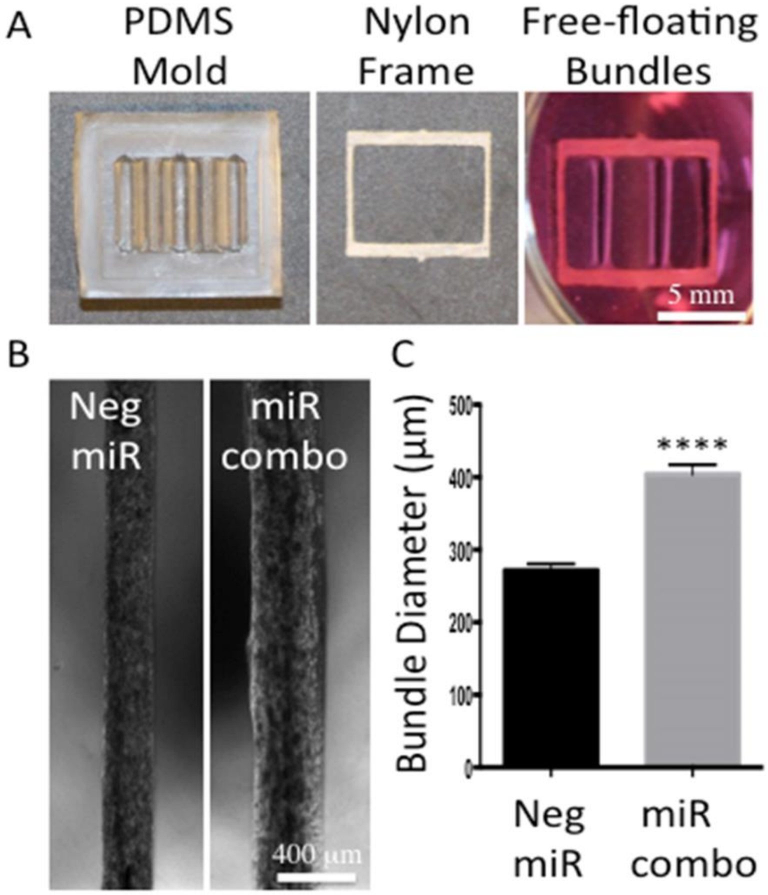

Supplementary Figure 2

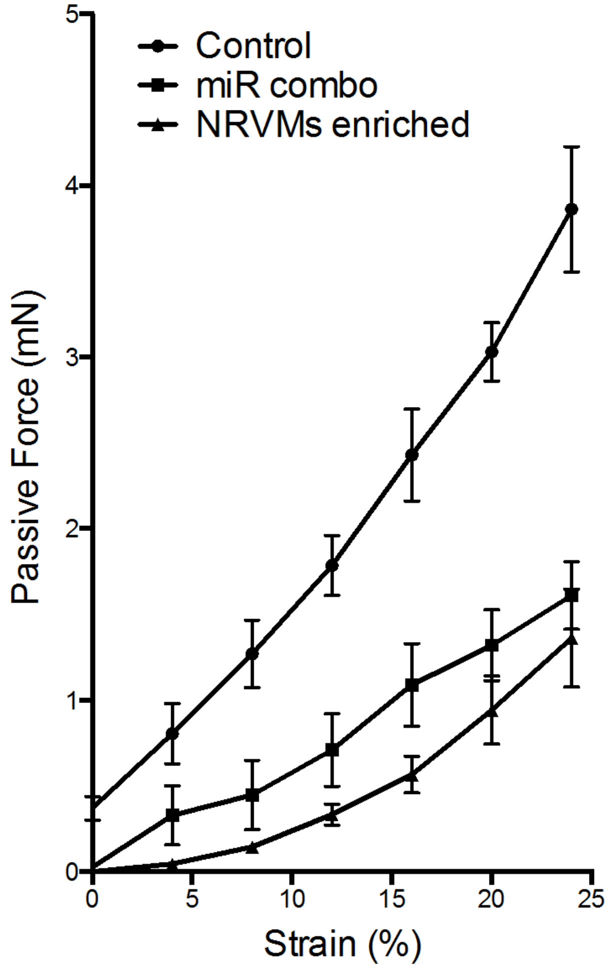

Supplementary Figure 3

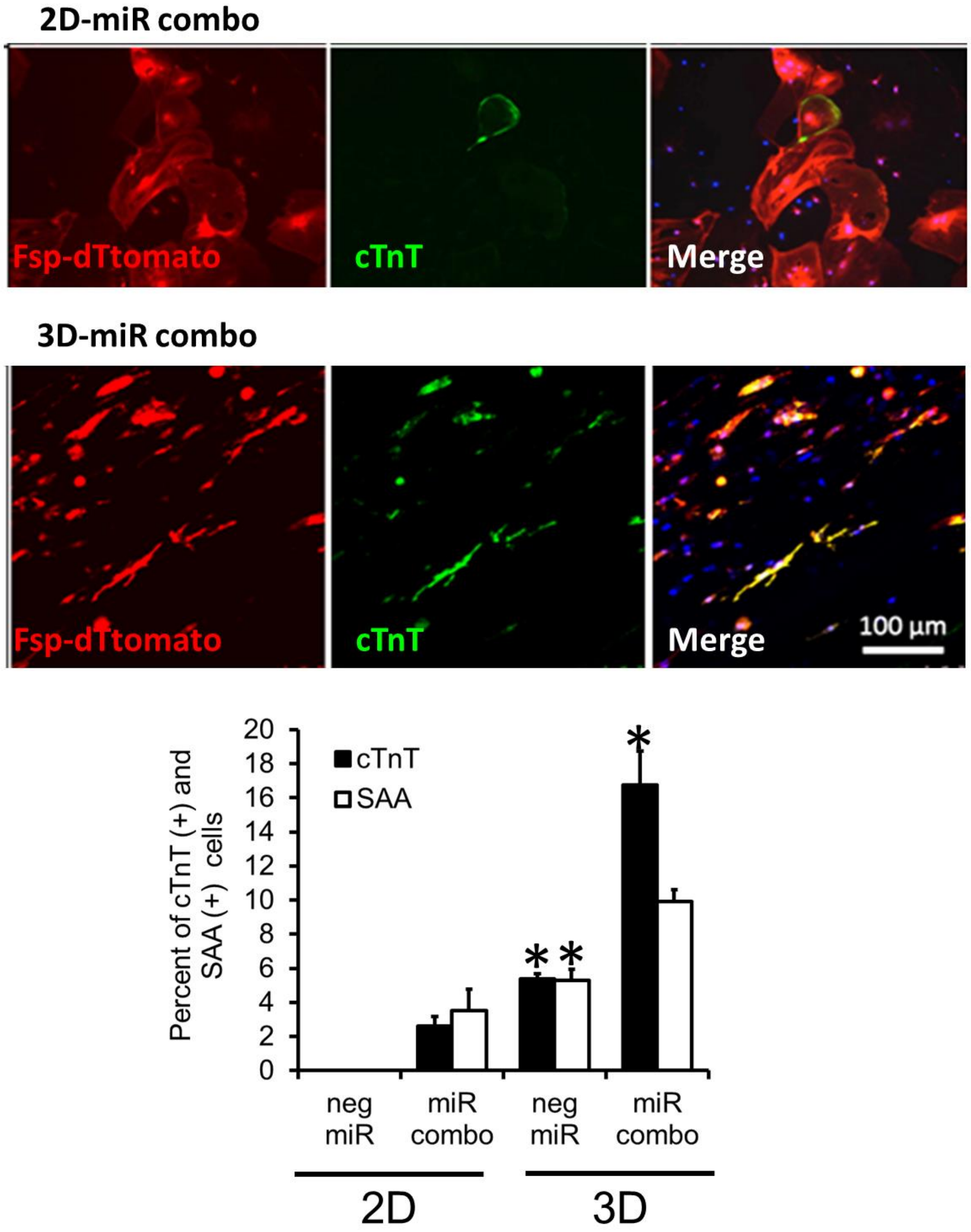

Supplementary Figure 4

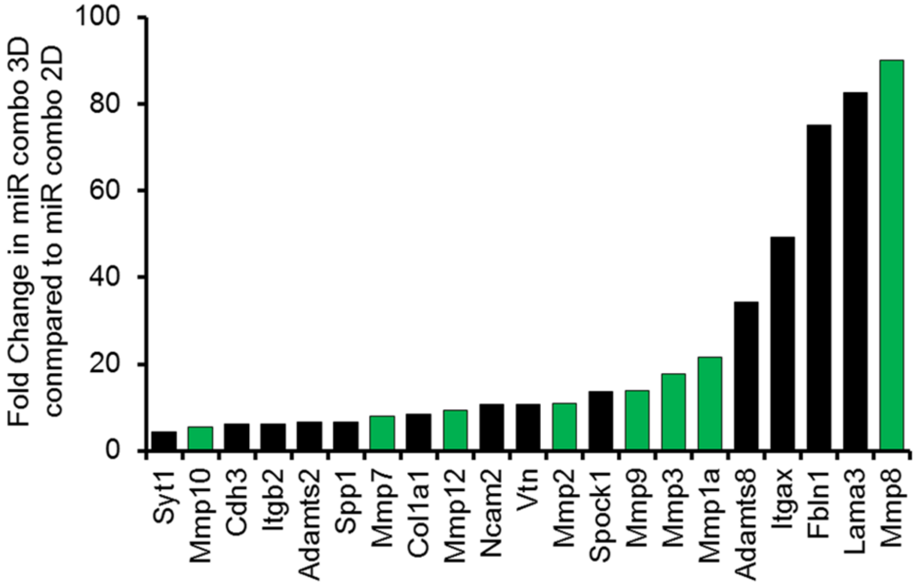

Gene Name	Fold change miR combo 3D vs miR combo 2D
Syt1	4.3517
**Mmp10**	**5.5094**
Cdh3	6.0892
Itgb2	6.2255
Adamts2	6.609
Spp1	6.6848
**Mmp7**	7.9427
Col1a1	8.4255
**Mmp12**	**9.2822**
Ncam2	10.5986
Vtn	10.5986
**Mmp2**	**10.9829**
Spock1	13.7507
**Mmp9**	**13.9811**
**Mmp3**	**17.7361**
**Mmp1a**	**21.5201**
Adamts8	34.1876
Itgax	49.3464
Fbln1	74.9954
Lama3	82.4821
**Mmp8**	**90.0245**

Supplementary Figure 5

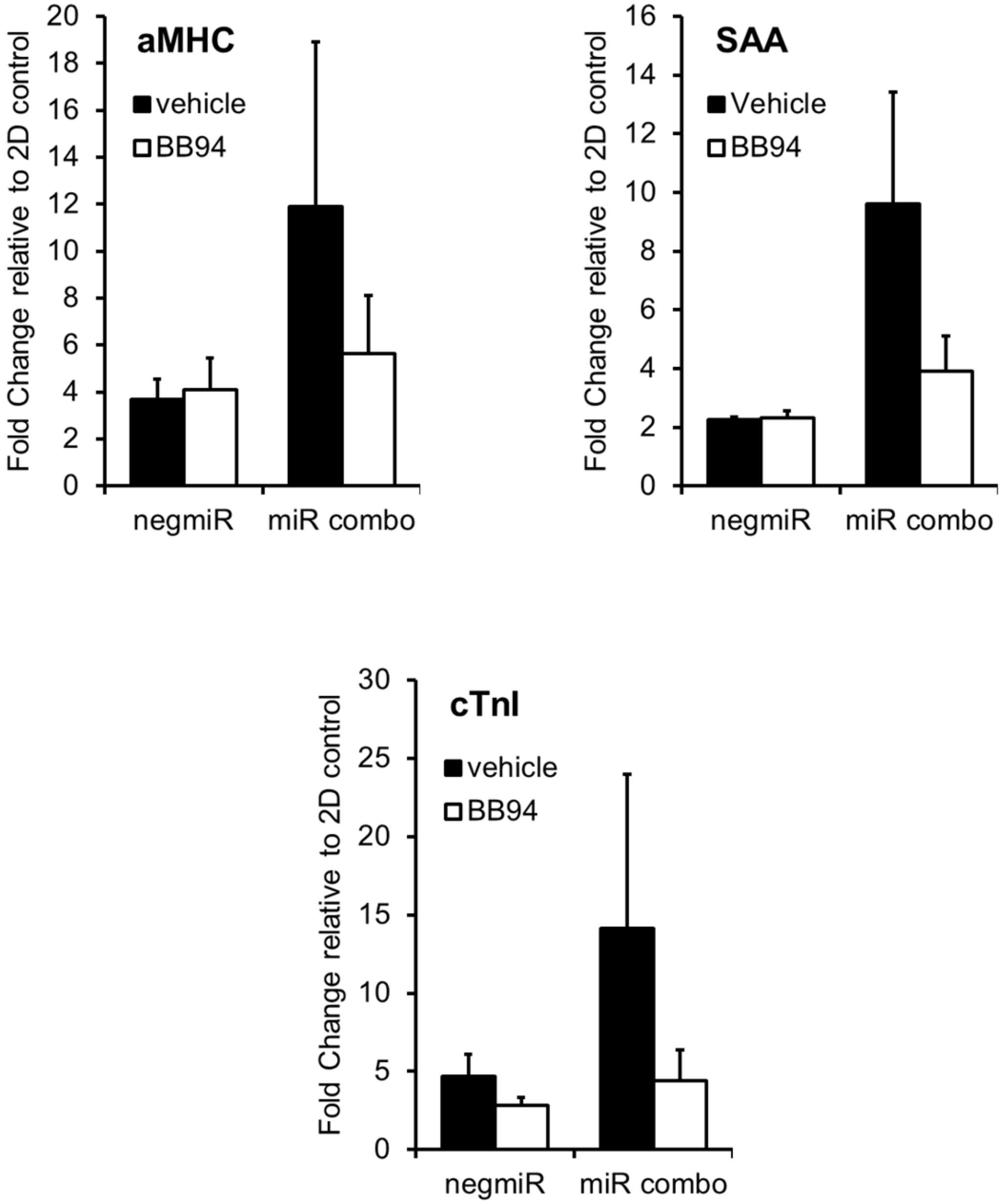

Supplement: Supplementary Information [file srep38815-s1.pdf]
